# Postural Stability in Adolescent Girls with Progressive Idiopathic Scoliosis

**DOI:** 10.1155/2019/7103546

**Published:** 2019-12-11

**Authors:** Marzena Wiernicka, Tomasz Kotwicki, Ewa Kamińska, Dawid Łochyński, Mateusz Kozinoga, Jacek Lewandowski, Piotr Kocur

**Affiliations:** ^1^Department of Musculoskeletal Rehabilitation, Faculty of Physical Education, Sport and Rehabilitation, Poznań University of Physical Education, 61-871 Poznań Królowej Jadwigi Street 27/39, Poland; ^2^Department of Spine Disorders and Pediatric Orthopedics, Poznan University of Medical Sciences, 61-544 Poznań, ul. 28 Czerwca 1956 r. Street 135/147, Poland

## Abstract

The aim of this work was to analyze postural stability of girls having progressive form of idiopathic scoliosis and undergoing specific period of the adolescent growth spurt. Twenty-seven girls, aged 13.4 ± 1.2 years, presenting structural idiopathic scoliosis, thoracic or thoracolumbar pattern, radiological Cobb angle 41.7 ± 17.4° (study group) and 37 healthy girls (control group) were included. The groups were sex, age, height, weight, and BMI matched. Postural stability examination was performed using two stabilometric platforms with visual control (eyes open) at three stages: (1) both legs' stance, (2) left leg stance, and (3) right leg stance. The Center of Pressure (COP) sway path length, the area and the displacement amplitude were compared. For the double stance, no difference in postural stability parameters between the groups was found. However, for the right leg stance, the total sway path length was longer (*p* = 0.04) and the mean amplitude of the lateral COP displacement was increased (*p* = 0.03) in the scoliotic group. In conclusion, for double stance, the adolescent girls with progressive form of idiopathic scoliosis revealed fair postural stability compared to control group. An impaired postural control was observed during right leg stance.

## 1. Introduction

Idiopathic scoliosis represents a developmental, structural, three-dimensional deformity of the spine and can affect functioning of other systems of the human body including musculoskeletal, nervous, cardio-respiratory and internal organ systems [1–6].

Postural stability is considered as an ability to maintain static body alignment in space and to restore the body balance, lost by the action of destabilizing factors [7]. Structural scoliosis has been claimed to be such a factor [7]. It has been indicated that severe deformation of the body morphology resulting from scoliosis could negatively influence the sensorimotor control of posture and decrease the postural balance capabilities. Alternatively, it has been hypothesized that scoliosis may result from the impaired postural stability [8, 9].

Before or after the pubertal period, the body morphology undergoes slow changes. Thus, the postural control can be more easily maintained. In opposition, during puberty, the rapid growth was postulated to be a trigger factor for scoliosis progression [10]. It is supposed that a relatively rapid change of body morphology as well as the longitudinal spine growth can impair the control of vertical posture.

The aim of the study was to compare the static postural stability among the healthy and scoliotic adolescent girls.

*Study Design.* Cross-sectional analysis of postural stability of adolescent girls presenting progressive form of idiopathic scoliosis versus healthy controls, sex, age, and BMI matched.

## 2. Material and Methods

### 2.1. Participants

**Figure 1:**
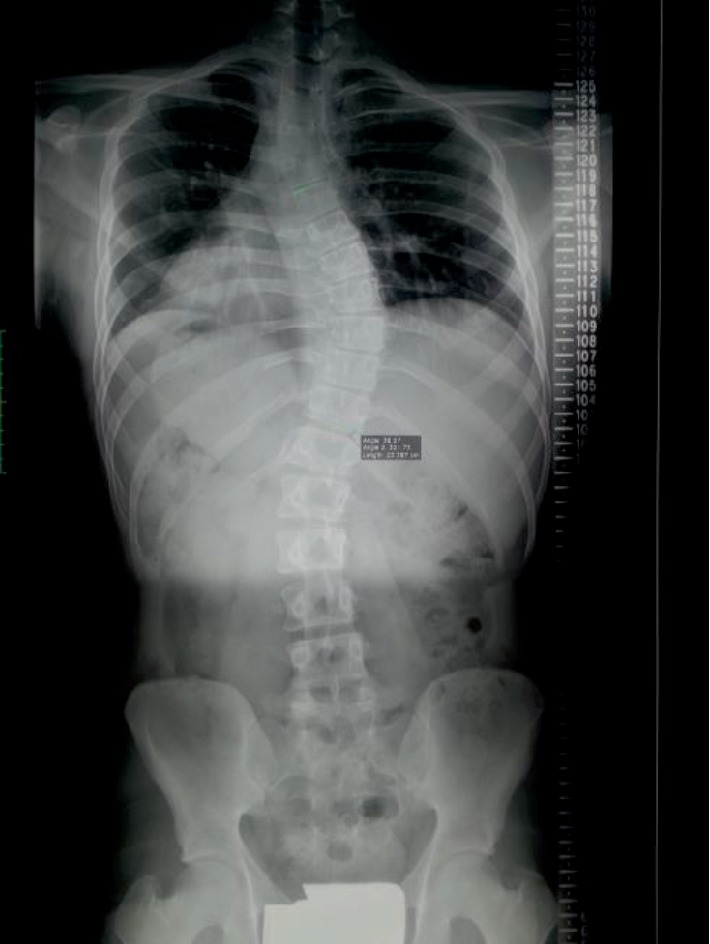
Standing P-A radiograph of 13-year-old girl with right thoracic idiopathic scoliosis. Cobb angle 38°. The girl was referred to consider treatment using corrective bracing.

All consecutive adolescent girls who presented to the outpatient clinic for nonsurgical treatment of idiopathic scoliosis within the 6 months period were analyzed in terms of inclusion to this study. The inclusion criteria were: female sex, age of 11–16 years; idiopathic scoliosis diagnosed on clinical examination and radiological measures; the Cobb angle over 20°; radiologically proven progression of the Cobb angle of at least 6° within the previous 6 months. All patients underwent radiological examination comprising a standing P-A long cassette X-ray of the spine (Figure 1). The recruitment of all participants to the study took 12 months.

Informed consent was obtained from the parents or caregivers for all patients. The study was approved by the Institutional Review Board of the Poznan University of Medical Sciences.

### 2.2. Data Acquisition

Participants underwent examination consisting of stability assessment using two stabilometric platforms (CQ Electronic System, Poland), one for each leg, and supplied with software dedicated to analyze postural stability.

### 2.3. Experimental Procedure

#### 2.3.1. Examination on Stabilometric Platform

The girls were asked to stand quietly on the two platforms. Three stages of examination were performed: (1) standing on both legs having the feet at the hip width apart; (2) standing on the left leg, and (3) standing on the right leg. For each condition, three trials were performed, separated with a 2-minute break. For each trial, the child was standing on the platform for 30 seconds with hands at the side, looking straight ahead and focusing gaze on the wall located approximately 1 m ahead. All trials were performed with the eyes open. Before each trial, the platforms were calibrated in the Cartesian coordinates system. The most stable trial (the one having the shortest Center of Pressure (COP).

#### 2.3.2. Data Analysis

Four parameters were analyzed to assess postural stability: (1) sway path—the length of the line outlined by the moving COP, measured in millimeters, (2) sway area—the enclosed area delineated by the COP during its oscillation within the base of support, expressed in square millimeters, (3) mean amplitude of the left and the right lateral COP displacement, expressed in millimeters, (4) mean amplitude of antero-posterior COP displacement, expressed in millimeters.

### 2.4. Statistical Analyses

The parameters were compared between the both groups, separately for each stage. Normality was checked using Shapiro–Wilk test. Since the data distribution for all parameters was not normal, the nonparametric Mann–Whitney *U* test was used for comparisons and the Spearman rank test for correlation. The *p* value ≤0.05 was considered significant.

## 3. Results

Twenty seven consecutive girls with idiopathic scoliosis fulfilling the inclusion criteria (study group) and 37 healthy girls (control group) were examined. There were 12 patients with single curve scoliosis (6 thoracic and 6 thoracolumbar) and 15 patients with double curve scoliosis. The mean Cobb angle of the primary curve was 41.7 ± 17.4°, range 22–90°. The patients had no previous treatment apart from unspecific exercises. The anthropometric data are shown in Table 1.

Postural stability parameters related to the COP sway, i.e., sway path and sway area did not differ between the groups either for double or single left leg stance. However, for the right leg stance, the girls with scoliosis revealed increased total COP sway path (Table 2). Similarly, the stability parameters related to the COP displacement did not differ for double or single left leg stance. However, the increased mean amplitude of latero-lateral COP displacement during right leg stance was observed in the scoliotic group (Table 3).

No significant correlation between the results of stabilometric examination and the following parameters: age (*p* > 0.05), Cobb angle (*p* > 0.05), or Risser (*p* > 0.05) sign was found. Also, no statistical relation between the stabilometric parameters and the following categories: pre-versus post-menarchial status (*p* > 0.05), thoracic versus lumbar curvature (*p* > 0.05) and single versus double curvature (*p* > 0.05) was found.

## 4. Discussion

This study aimed to evaluate static postural stability of girls with progressive idiopathic scoliosis in comparison to healthy girls. The stability was unimpaired during standing on both legs or left leg; however, it was decreased during right leg stance, as documented by increased values of the corresponding parameters which indicates deteriorated postural stability [6, 11].

No significant difference between scoliotic girls and the controls during double stance was revealed. This finding can be considered surprising in view of other studies which report decreased stability in girls with scoliosis [6, 9, 12–19]. In those studies, the angle of curvature was smaller (27 ± 6° [6]; 27.2 ± 12.4° [9]; 29.4 ± 9.4° [13]; 13.5 ± 5.5° [14], respectively) comparing to this study (41.7 ± 17.4°). It might be supposed that the postural system developed mechanisms of compensation or adaption to the altered body morphology.

Effectiveness of posture control depends on age in terms of maturity of the sensorimotor system [7, 20]. It is to note that several studies on scoliotic patients [3–4, 12] having reported an impaired postural control comprised the patients with larger age range (11–21 years) comparing to this study (11–16 years for both scoliotics and controls).

In the study by Dalleau et al. (2007), the impairment of postural stability during double leg stance was claimed to result from asymmetric control of the trunk while both morphological changes and sensorimotoric impairment were responsible for deformation. However, the groups of healthy and scoliotic girls were matched by weight but not by age, height, and BMI as they were in this study [13].

Park et al. (2013) revealed that comparing to healthy controls, the scoliotic teenagers revealed worse postural stability under visual control [19]. That study endorsed different methodology, since the participants were asked to shift their body to the specific targets under visual control while in this study the subjects were instructed to look straight ahead during quiet standing task [16].

The girls examined in this study (both the study and the control group) have been undergoing the period of rapid pubertal growth (11–13 years), considered the critical period of motor and sensory control of the musculoskeletal system. Thus, they could experience more difficulties in controlling postural balance. Moreover, in the study group, the progression of the curve itself was responsible for the important modification of the trunk morphology which can be considered a factor worsening postural control [21–25]. Nevertheless, the results of this study did not confirm previously reported postural impairment.

The postural stability was decreased during right leg stance. Previous studies did not evaluate the stability during standing on one single leg in girls with scoliosis. The only one reporting on single leg stance stability by Simoneau et al. (2006) pointed out that disturbance of the ankle proprioception contributed to decreased stability in scoliotic individuals [3, 4]. The reasons of decreased postural stability during right leg stance found in this study are unclear. In the right–handed persons, the left lower limb has the principal supportive role. It is worth to note that 17 out of 27 thoracic curvatures were right convex [13, 14], which could affect the loading on the right lower limb. Another question requiring further studies concerns the dynamics of compensation by secondary spinal curvatures which develop over time. The findings cannot be explained solely by the side of curvature. Therefore, other factors contributing to decreased stability of girls with idiopathic scoliosis when standing on the right foot are to be searched.

## 5. Clinical Rehabilitation Impact

Rapid change of body morphology related to puberty as well as the longitudinal growth spurt of the spine can impair the control of the vertical posture while it coincidences with rapid deterioration of scoliotic spinal curvature. These findings suggest considering postural stability techniques in the rehabilitation of adolescent girls with progressive idiopathic scoliosis.

## 6. Conclusion

Adolescent girls with structural progressive idiopathic scoliosis revealed fair postural stability when assessed with stabilometric technique during quiet double stance. An impaired postural control was detected at right leg stance for girls with progressive form of idiopathic scoliosis compared to able bodied girls' age-, weight-, height-, and BMI-matched.

## Figures and Tables

**Table 1:** Anthropometric data for the study and control group.

Parameter	Study group	Control group	*p*
	*n* = 27	*n* = 37	Unpaired *t*-test
Age (years)	13.4 ± 1.2	13.2 ± 1.6	0.3550
	(11.0−16.0)	(11.0−16.0)	
Height (cm)	160 ± 7.5	159 ± 7.0	0.3338
	(141−175)	(148−172)	
Weight (kg)	49.3 ± 8.8	48.5 ± 8.8	0.7237
	(31.1−63.5)	(30.0−64.0)	
BMI	19.2 ± 2.8	19.1 ± 2.8	1.000
	(15.2−25.1)	(13.7−26.7)	

The values of all parameters are presented as mean ± standard deviation followed by minimum and maximum in brackets.

**Table 2:** Sway path and sway area in the study and the control group.

Parameter	Study group	Control group	*p*-value
	*n* = 27	*n* = 37	(Mann–Whitney *U* test)
Sway path (double stance) [mm]	418.4 ± 92.6	400.7 ± 90.5	0.4147
	(235–648)	(251–598)	
Sway path (left foot stance) [mm]	1200.4 ± 310.0	1125.7 ± 364.4	0.2212
	(632–1886)	(587–1841)	
Sway path (right foot stance) [mm]	1233.9 ± 278.3	1094.6 ± 300.3	**0.0408**
	(695–1758)	(543–1952)	
Sway area (double stance) [mm^2^]	571.3 ± 278.8	513.0 ± 224.2	0.4384
	(174–1342)	(140–1027)	
Sway area (left foot stance) [mm^2^]	3020.7 ± 1253.2	3049.6 ± 1805.4	0.6150
	(781–5528)	(901–10346)	
Sway area (right foot stance) [mm^2^]	3367.5 ± 1415.9	2894.2 ± 1520	0.1000
	(1184–6896)	(948–7707)	

The values of all parameters are presented as mean ± standard deviation followed by minimum and maximum.

**Table 3:** Mean amplitude lateral sway and mean amplitude antero-posterior sway values.

Parameter	Study group	Control group	*p*-value
	*n* = 27	*n* = 37	(Mann–Whitney *U* test)
Mean amplitude antero-posterior sway (double stance) [mm]	3.5 ± 1.4	3.1 ± 1.1	0.3722
	(1.5–6.6)	(1.2–6.1)	
Mean amplitude antero-posterior sway (left foot stance) [mm]	5.4 ± 1.3	5.8 ± 1.7	0.6462
	(2.6–7.6)	(2.5–9.5)	
Mean amplitude antero-posterior sway (right foot stance) [mm]	5.6 ± 1.6	5.7 ± 1.7	0.8396
	(3.4–10.2)	(2.8–9.4)	
Mean amplitude lateral sway (double stance) [mm]	1.5 ± 0.7	1.6 ± 0.6	0.7665
	(0.4–3.9)	(0.6–3.2)	
Mean amplitude lateral sway (left foot stance) [mm]	3.7 ± 0.9	3.8 ± 1.2	0.7665
	(2.0–5.5)	(1.9–7.2)	
Mean amplitude lateral sway (right foot stance) [mm]	4.1 ± 1.1	3.7 ± 1.2	**0.0310**
	(2.2–6.6)	(2.0–7.1)	

The values of all parameters are presented as mean ± standard deviation followed by minimum and maximum in brackets.

## Data Availability

The data used to support the findings of this study are available from the corresponding author upon request.

## Additional Points

*Highlights.* (i) Postural stability of girls with scoliosis was compared to matched controls. (ii) Patients were in specific period of progression at pubertal growth spurt. (iii) Right leg stance revealed postural impairment in scoliotic group.

## References

[1] Herman R., Mixon J., Fisher A., Maulucci R., Stuyck J. (1985). Idiopathic scoliosis and the central nervous system: a motor control problem. *Spine*.

[2] Zabjek K. F., Leroux M. A., Coillard C., Rivard C.-H., Prince F. (2005). Evaluation of segmental postural characteristics during quiet standing in control and idiopathic scoliosis patients. *Clinical Biomechanics*.

[3] Simmoneau M., Richer N., Marcier P., Allard P., Teasdale N. (2006). Sensory deprivation and balance control in idiopathic scoliosis adolescent. *Experimental Brain Research*.

[4] Simoneau M., Mercier P., Blouin J., Allard P., Teasdale N. (2006). Altered sensory-weighting mechanisms is observed in adolescents with idiopathic scoliosis. *BMC Neuroscience*.

[5] Zabjek K. F., Coillard C., Rivard C.-H., Prince F. (2008). Estimation of the centre of mass for the study of postural control in idiopathic scoliosis patients: a comparison of two techniques. *European Spine Journal*.

[6] Gruber A. H., Busa M. A., Gorton III G. E., Van Emmerik R. E. A., Masso P. D., Hamill J. (2011). Time-to-contact and multiscale entropy identify differences in postural control in adolescent idiopathic scoliosis. *Gait & Posture*.

[7] Błaszczyk J. W. (2004). *Clinical Biomechanics*.

[8] Yamamoto H., Yamada K. (1976). Equilibrial approach to scoliotic posture. *Agressologie*.

[9] Beaulieu M., Toulotte C., Gatto L., Rivard C.-H., Simmoneau M., Allard P. (2009). Postural imbalance in non-treated adolescent idiopathic scoliosis at different periods of progression. *European Spine Journal*.

[10] Zaina F., De Mauroy J., Grivas C. T. (2014). Bracing for scoliosis in 2014: state of the art. *European Journal of Physical and Rehabilitation Medicine*.

[11] Wollacott M. H., Shumway-Cook A., Nashner L. M. (1986). Aging and posture control: changes in sensory organization and muscular coordination. *The International Journal of Aging and Human Development*.

[12] Chen P. Q., Wang J. L., Tsuang Y. H., Liao T. L., Huang P. I., Hang Y. S. (1998). The postural stability control and gait pattern of idiopathic scoliosis adolescent. *Clinical Biomechanics*.

[13] Dalleau G., Allard M. S., Beaulieu M., Rivard C.-H., Allard P. (2007). Free moment contribution to quiet standing in able-bodied and scoliotic girl. *European Spine Journal*.

[14] Dalleau G., Damavandi M., Leroyer P., Verkindt C., Rivard C.-H., Allard P. (2011). Horizontal body and trunk center of mass offset and standing balance in scoliotic girl. *European Spine Journal*.

[15] Silferi V., Rougier P., Labelle H., Allard P. (2004). Postural control and idiopathic scoliosis: comparison between healthy and scoliotic subject. *Revue de Chirurgie Orthopédique et Réparatrice de l'appareil Moteur*.

[16] Park J.-Y., Park G. D., Lee S.-G., Lee J.-C. (2013). The effect of scoliosis angle on centre of gravity sway. *Journal of Physical Therapy Science*.

[17] Gregoric M., Pecak F., Trontej J. V., Dimitrijević M. R. (1981). Postural control scoliosis: a statokinesimetric study in patients with scoliosis due to neuromuscular disorders and in patients with idiopathic scoliosis. *Acta Orthopaedica Scandinavica*.

[18] Byl N. N., Gray J. M. (1993). Complex balance reactions in different sensory conditions: adolescents with and without idiopathic scoliosis. *Journal of Orthopaedic Research*.

[19] Natul M. L., Allard P., Hinse S. (2002). Relations between standing stability and body posture parameters in adolescent idiopathic scoliosis. *Spine*.

[20] Wolański N. (2012). *Human Biological Development*.

[21] Allum J. H. J., Bloem B. R., Carpenter M. G., Hulliger M., Hadders-Algra M. (1998). Proprioceptive control of posture: a review of new concepts. *Gait & Posture*.

[22] Buchanan J. J., Horak F. B. (1999). Emergence of postural patterns as a function of vision and translation frequency. *Journal of Neurophysiology*.

[23] Hunter M. C., Hoffmann M. A. (2001). Postural control: visual and cognitive manipulation. *Gait & Posture*.

[24] Iqbal K., Pai Y.-C. (2000). Predicted region of stability for balance recovery: motion at the knee join can improve termination of forward movements. *Journal of Biomechanics*.

[25] Lo Monarco E. A., Paquet N., Hui-Chan C. W. Y. (2004). Responses to whole head-and-body tilts with and without passive ankle dorsiflection in the absence of visual feedback. *Clinical Biomechanics*.

